# A Microbial-Centric View of Mobile Phones: Enhancing the Technological Feasibility of Biotechnological Recovery of Critical Metals

**DOI:** 10.3390/bioengineering12020101

**Published:** 2025-01-22

**Authors:** Chiara Magrini, Francesca Verga, Ilaria Bassani, Candido Fabrizio Pirri, Annalisa Abdel Azim

**Affiliations:** 1 Politecnico di Torino, Department of Environment, Land and Infrastructure Engineering (DIATI), 10129 Turin, Italy; chiara.magrini@polito.it (C.M.); francesca.verga@polito.it (F.V.); 2 Centre for Sustainable Future Technologies, Fondazione Istituto Italiano di Tecnologia, 10144 Turin, Italy; ilaria.bassani@iit.it (I.B.); fabrizio.pirri@iit.it (C.F.P.); 3 Politecnico di Torino, Department of Applied Science and Technology (DISAT), 10129 Turin, Italy

**Keywords:** e-waste, bioleaching, mobile phone

## Abstract

End-of-life (EoL) mobile phones represent a valuable reservoir of critical raw materials at higher concentrations compared to primary ores. This review emphasizes the critical need to transition from single-material recovery approaches to comprehensive, holistic strategies for recycling EoL mobile phones. In response to the call for sustainable techniques with reduced energy consumption and pollutant emissions, biohydrometallurgy emerges as a promising solution. The present work intends to review the most relevant studies focusing on the exploitation of microbial consortia in bioleaching and biorecovery processes. All living organisms need macro- and micronutrients for their metabolic functionalities, including some of the elements contained in mobile phones. By exploring the interactions between microbial communities and the diverse elements found in mobile phones, this paper establishes a microbial-centric perspective by connecting each element of each layer to their role in the microbial cell system. A special focus is dedicated to the concepts of ecodesign and modularity as key requirements in electronics to potentially increase selectivity of microbial consortia in the bioleaching process. By bridging microbial science with sustainable design, this review proposes an innovative roadmap to optimize metal recovery, aligning with the principles of the circular economy and advancing scalable biotechnological solutions for electronic waste management.

## 1. Introduction

The information and communication technology (ICT) sector, encompassing devices like personal computers, laptops, smartphones, and tablets, along with its digital infrastructure like data centers and communication networks, is projected to account for 14% of the global carbon footprint by 2040. Mobile phones exemplify a product category characterized by a premature short lifespan [1]. Approximately 84% of the population in the world owns a smartphone. In 2022, there were almost 16 billion mobile phones worldwide, of which 5.3 billion became waste. In the EU, only 10% of individuals recycled their old mobile phones when replacing them, while 17% opted to give away or sell their devices. Notably, nearly half of the population retained their old phones within their households [2]. Mobile phone production is expected to reach 18 billion units by 2025. Despite the extensive research conducted in recent years to highlight the adverse environmental effects of intensive manufacturing, the smartphone industry remains primarily profit-oriented [3]. From raw material extraction to end of life (EoL), the life cycle of mobile phones increases the consumption of land and water [4]. A Deloitte report estimated that mobile phones contributed approximately 146 million tonnes of CO_2_ emissions in 2022 globally [5]. Raw material extraction and transport account for 83% of the emissions, while the data usage (i.e., electricity consumption) during the first year accounts for 11%.

In this context, the European Union (EU) is driving the transition to a circular economy through initiatives like the 2024/884/EU Directive on Ecodesign and the Critical Raw Materials Act, which aims to secure sustainable raw material supplies and achieve a 25% recycling rate for critical raw materials (CRMs) [6,7]. Mobile phones, with their complex design and an average of 62 recoverable metals, require tailored recycling solutions [8]. The development of technologies enabling the efficient extraction and recovery of secondary raw materials from EoL mobile phones is central in the circular economy framework. Pretreatment steps such as dismantling are essential for selective recovery of components [9,10,11].

Common methods for recovering metals include pyrometallurgy and hydrometallurgy. Pyrometallurgy is based on thermal treatment at temperatures ranging between 600 and 1200 °C. This technique is energy-intensive, produces corrosive acids, and lacks selectivity; thus, it is used for bulk recovery of base metals and the concentration of precious metals [12]. Hydrometallurgy, where the technologies of aqueous solutions are applied for the recovery of metals, prevails as the dominant recycling method due to the higher economic viability, environmental sustainability, and accuracy compared to pyrometallurgy for precious and critical metals. However, drawbacks such as acid toxicity, heavy metal pollution, and sludge generation were highlighted [13]. Among greener technologies, biohydrometallurgy emerges as a promising candidate due to the mild conditions required and its sustainable intrinsic nature. This technique, often identified as bioleaching, is defined as the green extraction of metals through microorganism metabolic activity or metabolic compounds [14,15,16]. In the present work, we review the most recent bioleaching studies, giving special attention to those focusing on mobile phone recycling through the exploitation of microbial consortia rather than commonly used single-cell cultures.

The present review aims to spotlight the importance of transitioning from research based on single-material recovery approaches to comprehensive, holistic recovery strategies. Our work also highlights the insufficient studies on mobile phone-specific recycling through bioleaching, particularly the scarcity of research focusing on components other than PCBs. Furthermore, this review examines the interactions between microbial communities and the primary elements found in mobile phones, aiming to establish a microbial-centric perspective that views end-of-life (EoL) mobile phones as potential sources of valuable metals exploitable through processes such as biohydrometallurgy. Despite this potential, empirical evidence remains limited because most studies concentrate on the recycling of entire mobile phones without selectively separating individual components or only with a predominant focus on PCBs and batteries, while few deal with screens, and camera and casing fractions. Additionally, emerging concepts like ecodesign and modular design in mobile phones and electronics are explored as fundamental factors that could parallelly improve the selectivity and scalability of biotechnological metal recovery from EoL devices in the near future.

## 2. What’s Inside Smartphones?

Based on a recent study, the main components inside smartphones are metal, plastic, and glass, representing 45%, 17%, and 32% wt., respectively [17].

**Figure 1:**
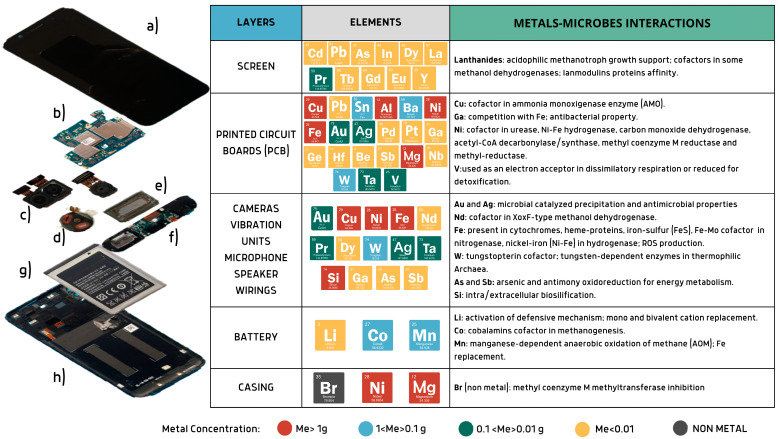
Overview of smartphone components and raw material content. Metal concentrations are based on [17]. Smartphone elements: (**a**) screen, (**b**) printed circuit board, (**c**) cameras, (**d**) vibration units, (**e**,**f**) speaker units, (**g**) Li-ion battery, (**h**) case. Created via Canva.com (accessed on 23 September 2024).

Of the 83 stable elements of the periodic table, at least 70 can be found in mobile phones, and on average 62 of them contribute to making them ‘smart’. Figure 1 shows the individual components of a smartphone model with the corresponding elemental content and biological functions in microorganisms. Among these elements, eleven metals (i.e., Au, Pd, Ni, Cu, Si, Mg, Pt, Nd, Al, Sn, Fe) define 97% of the total pure metal value of a single average smartphone [17]. Gómez et al., 2023, characterized and described the composition of cross-generational EoL mobile phones (*feature phones*, *multimedia phones*, *smartphones*), providing a comprehensive view of the evolution of the different components, with a correlation between the increasing use of critical materials and the improved performances achieved by those devices [18]. The concentration of materials in mobile phones, including critical and/or hazardous/high-value elements, rare earth elements (REEs), and platinum group metals (PGMs), was found to be at least twice and, in some cases, up to 600 times higher than their corresponding natural ores. Hence, EoL mobile phones are considered an exploitable mine of great intrinsic value in the circular economy.

### 2.1. Natural Microbe–Metal Interaction: A Focus on Elements Contained in Mobile Phones Layer by Layer

**Figure 2:**
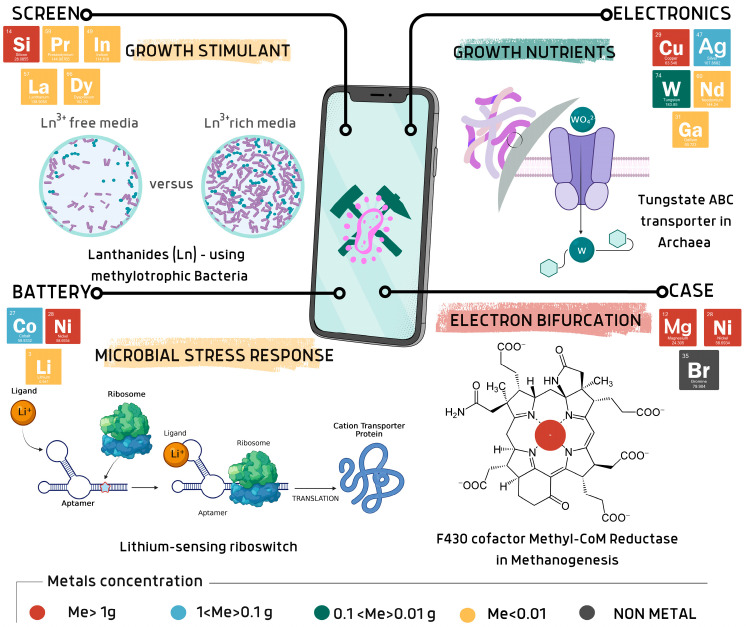
Examples of natural interactions among microbes and corresponding metals contained in mobile phones. Created via Canva.com (accessed on 23 September 2024).

The next sections aim to provide a microbial-centric view of smartphones, mapping all the relevant metals contained in each layer based on the most recent studies. Studies on specific interactions between microorganisms and metals are herein discussed and summarized in Table 1. Microorganisms require only a selected subset of the atomic elements; Kaleigh A. et al., 2022, in their work, presented a real biocentric tour of the periodic table analyzing the biological utility of each element [19]. Given the great heterogeneity of the elements contained in EoL mobile phones, it is essential to analyze the exact composition of the layers in order to predict the possible interactions between microorganisms and the electronic components. In fact, even if not all metals are essential for life, they can still play an important role by interacting directly with microbes or indirectly with the products of their metabolism. Figure 2 represents some of the most relevant examples of metal utilization observed in nature by the microbial world, which could be exploited in EoL MP recycling strategies, and which are discussed in the following sections.

Vibration units in MP contain tungsten (W), which is a key element in the growth of methanogens [20]. Screens are rich in lanthanides (Ln), which stimulate the growth of methanotrophic bacteria and whose uptake in culture media is favoured over calcium [21]. Nickel, which is one of the most abundant metals in mobile phones, is also an essential element for several microorganisms. For instance, the role of nickel is widely recognized in the redox metabolism of methanogens. Batteries in mobile phones contain, among the other metals, lithium at the cathodic pole. Some bacteria can respond to the lithium presence by activating the expression of cation transporter genes, which are involved in osmoregulation by the lithium riboswitches [22]. Those are only some of the elements that we can find in mobile phones, and which possibly are important for microbial life and whose interaction could be strategic for bioleaching applications.

#### 2.1.1. Casing

The content of plastic in mobile phones varies depending on the generation. According to Bruno, M. et al., 2022 [23], plastic content in smartphones is 37% wt., with a general decreasing trend for plastic in favor of metal components. Commonly used plastics in mobile phones are polycarbonate (PC), acrylonitrile butadiene styrene (ABS), polyethylene (PE), and polypropylene (PP). Among them, PC is largely used in cases and internal parts because of its mechanical resistance and optical transparency properties. PE and PP are applied as electric insulating material in wires and cables, battery covers, and protective cases for the most delicate parts (e.g., circuits). Bioplastics like polylactide (PLA) can be a valid alternative to the above-mentioned fossil-based plastics, due to their biodegradable nature, though their application to electronic devices is still limited. Among non-metals, bromine (Br) is usually found as a flame retardant in plastics. Brominated flame retardants (BFRs) have been extensively used in electrical and electronic equipment as they are considered the most effective ones [24]. The use of BFRs in the latest generation of smartphone is progressively decreasing due to their toxicity. Among metals, nickel (Ni) and magnesium (Mg) alloys are used to reduce electromagnetic interference (EMI) in cases.

##### Bromine

Bromine (Br) is known to be essential only for animals, but not for prokaryotes. Halogenated methane analogs, such as bromoform (CHBr_3_), are proposed to inhibit methanogenesis by competitively binding with key enzymes, such as methyl coenzyme M methyltransferase [25]. Recent studies are reporting the effect of using bromoform containing seaweed *Asparagopsis* in ruminants’ feed for the reduction in methane production. The effect is linked to halogenated methane analog (HMA) components of *Asparagopsis*, which inhibit key steps involved in methanogenesis [26].

#### 2.1.2. Printed Circuit Board (PCB)

PCBs are, along with magnets, the most important components of smartphones in terms of metals. PCBs, whose fabrication represents the main source of greenhouse gas emissions, contains a high concentration of ferrous (iron, steel, and nickel) and nonferrous (copper) metals [27]. According to Gomez et al., 2023, copper (Cu) accounts for roughly 62% wt. of metals in PCBs [18]. In general, the copper amount is observed to increase through the generations of mobile phones due to the increased need of larger and more conductive PCBs. Lead (Pb) and tin (Sn) are the other two key metals present in this layer. Other base metals comprise aluminum (Al), barium (Ba), nickel (Ni), and iron (Fe). Among platinum group metals (PGMs), we can find gold (Au), silver (Ag), palladium (Pd), and platinum (Pt) as part of connectors between wires and PCB, due to their excellent conductive properties and their good thermal stability. REEs are also present in PCBs, accounting for 2.6% of the total PCB weight. Neodymium (Nd) and erbium (Er) were found at remarkable concentrations (on average, 1.42 mg/g and 9.18 mg/g, respectively). This can be attributed to the use of REEs in microphones and vibrators because of their magnetic properties. Furthermore, PCBs contain other CRMs, such as gallium (Ga), germanium (Ge), hafnium (Hf), beryllium (Be), antimony (Sb), magnesium (Mg), niobium (Nb), tungsten (W), tantalum (Ta), and vanadium (V) [18].

##### Copper

Copper is considered essential for many organisms within archaea, Eukarya, and bacteria domains, although an excess of copper is toxic [28]. As a trace element, copper has an important role in cellular function like other transition metals. Its ability to undergo redox changes makes copper an ideal cofactor in enzymes catalyzing electron transfers. Throughout evolution, bacteria and archaea have developed a highly regulated balance in copper metabolism [29]. The study by Andreini et al., 2008, deeply investigated the occurrence of copper in proteins through the three domains of life [30]. A proteome containing copper is scarce in prokaryotes due to both poorer bioavailability (with respect to iron in the ancient world) and the complexity of copper chemistry and the risks associated with it, which may have adversely affected the natural selection of copper-binding proteins. Copper plays a crucial role in the enzymes of Ammonia-Oxidizing Archaea (AOA), particularly in the ammonia monooxygenase (AMO) enzyme responsible for the first step of ammonia oxidation, converting ammonia to hydroxylamine [31]. AOA, including *Nitrososphaera viennensis*, rely on copper-dependent enzymes for their ammonia oxidation process. The presence of copper-dependent enzymes in AOA, like AMO, suggests that AOA have evolved specific mechanisms to acquire and utilize copper in their metabolic processes.

##### Gallium and Germanium

Gallium (Ga) and germanium (Ge) are not known for having beneficial or nutrient functions in biology, though both are considered non-toxic by the Royal Society of Chemistry. Few and pioneering studies are available in the literature on the microbial interaction with these elements [32]. However, a competing role of Ga with iron in biological systems is known [33]. Compounds containing Ga are usually used as antimicrobial and antibacterial agents inhibiting bacterial proliferation by disturbing the activity of iron-containing enzymes, such as deoxyribonucleotide reductase [34].

##### Nickel

Nickel (Ni) is known to be beneficial for many bacteria and archaea, being involved in a variety of cellular processes. Ni enzymes are found in 83% of anaerobic bacterial and archaeal proteomes, being detected in urease, Ni-Fe hydrogenase, carbon monoxide dehydrogenase, acetyl–CoA decarboxylase/synthase, methyl coenzyme M reductase, certain superoxide dismutases, some glyoxylases, acireductone dioxygenase, and methylene diurease [35]. Ni is also an essential cofactor for methanogens since it is incorporated into cofactor F_430_, a yellow chromophore of the methyl Co-M reductase (MCR) enzyme, which catalyzes the last reaction of methanogenesis and the first step of the anaerobic oxidation of methane [36].

##### Gold and Silver

Silver (Ag) and gold (Au) belong to PGMs and are considered of technological relevance. Neither of them have known biological roles; they are thought to be non-toxic and subjected to biotransformation by microbes, for example, in the synthesis of metal nanoparticles or in the bacterial production of metal(loid) nanostructures [37,38]. Previous research suggests that bacteria and archaea are directly involved in the different steps of the biogeochemical cycle of gold, from the formation of primary mineralization in hydrothermal and deep subsurface systems to its solubilization, dispersion, and re-concentration as secondary gold under surface conditions. Some thermophilic and hyperthermophilic bacteria and archaea (such as *Thermotoga maritime* and *Pyrobaculum islandicum*) were observed to be able to catalyze the precipitation of gold, leading to the formation of gold and silver sediments in New Zealand’s hot spring systems [39]. However, at certain concentrations, the nanoparticles or ionic forms of Ag and Au have antimicrobial properties, leading to cell death through alterations of cell morphology, interference with DNA replication, and oxidative stress induction [40,41].

##### Vanadium, Niobium, Tantalum

Niobium (Nb), tantalum (Ta), and vanadium (V) are all transition metals. Among them, only vanadium is known to have a biological role in some species, remaining toxic at high concentrations. For example, some bacteria can reduce vanadium, as a way of detoxification, or use it as an electron acceptor in dissimilatory respiration. Zhang et al., 2014, first referred to methanogenic archaeal species capable of reducing vanadium [42]. The strains *Methanosarcina mazei* and *Methanothermobacter thermautotrophicus* could reduce up to 10 mM and 5 mM of vanadate V^5+^ to vanadyl V^4+^, respectively, inducing solid extracellular precipitation. However, methanogenesis stops as vanadyl generation proceeds, possibly due to the redirection of electrons from methanogenesis to vanadate reduction. Conversely, neither Nb nor Ta have so far grasped biological relevance [19].

#### 2.1.3. Screen

Screen or liquid crystal display (LCD) composition evolved a lot through different generations of phones with a noticeable reduction in hazardous materials, such as cadmium (Cd) and lead (Pb), and the complete removal of the toxic metal arsenic (As) [18]. Screens can be considered a valuable source of critical metals like indium (In) in the form of indium tin oxide (ITO), used in conductive electrodes for its high conductivity and transparency. Cd, Pb, and In have no documented beneficial role in biology. The display contains a lot of rare earth elements (REEs) along which are lanthanum (La), praseodymium (Pr), europium (Eu), gadolinium (Gd), terbium (Tb), dysprosium (Dy), and yttrium (Y). A small amount of REEs is used to provide colors and brightness to the LCD. It is worthwhile to mention that LCD also contains plastic polymers, such as PC, polyethylene terephthalate (PET), and poly methyl methacrylate (PMMA).

##### Lanthanides and REEs

Lanthanides have only recently gained interest in the physiology of certain microorganisms, such as the methylotrophic strains. Over the last ten years, there has been a notable exploration and thorough investigation of bacteria utilizing lanthanides as cofactors of methanol dehydrogenases (MDHs) [43]. The evidence of the beneficial role of lanthanides for methylotrophs became evident when the acidophilic methanotroph *Methylacidiphilum fumariolicum* SolV, isolated from a volcanic mud pot and only possessing a XoxF-type methanol dehydrogenase, was unable to grow in laboratory cultures without the addition of mud pot water rich in lanthanides to the growth medium [44]. From these bacteria, researchers have identified protein lanmodulin, exhibiting exceptional selectivity and strong affinity for lanthanides, even where they are much less abundant than other essential metals like calcium [21,45]. In general, lanthanide-dependent enzymes seem to prefer the lighter lanthanides such as lanthanum, cerium, praseodymium, and neodymium (Nd), because an experiment conduced on methanotrophic/methylotrophic strains using a medium supplemented with heavier lanthanides showed a slower growth [46]. A protein found naturally in a bacterium (*Hansschlegelia quercus*) isolated from English oak buds exhibited strong capabilities to differentiate between rare earths. Harnessing its power could revolutionize all tech sectors by fundamentally changing how critical minerals like rare earths are harvested [47].

#### 2.1.4. Cameras, Speakers, and Vibration Units

The camera constitutes lens that direct light into the camera, a sensor that converts photons of light into an electrical signal, and software converting electrical signals into pictures. Both plastic and metallic parts are found in this layer. The metal fraction in cameras, accounting for 18.7% wt. [18], is associated with the connector and the sensor integrated into the silicon-made circuit framework. Smartphones’ camera contains a considerable amount of gold and silver, more than 1 mg and 0.9 mg per gram of cameras, respectively [18]. Speaker units in smartphones contain an outstanding concentration of Nd (about 85.5 mg per gram of speaker) that, blended with iron (Fe) and boron (B), makes the strongest permanent magnets. Despite its abundance, the Nd recycling rate is only 3% [48]. Due to the same features of magnetism, speakers also contain a high concentration of some REEs in the form of alloys, particularly praseodymium (Pr) and dysprosium (Dy) in concentrations of 8.8 and 5.6 mg/g of the speaker [18]. The vibration units contain mainly tungsten (W), which is CRM, and whose concentration increased cross-generationally, achieving 1.7 mg per gram of the vibration motor in smartphones.

##### Iron

Iron is the single most abundant element on Earth, also being involved in the biogeochemical cycling of other elements. Ferrous iron (Fe^2+^) is crucial for meeting the metabolic needs of microorganisms, especially in anoxic and acidic environments like those found in hosts, soils, aquatic environments, and waste sites. In aerobic conditions, Fe^2+^ is oxidized, contributing to the production of reactive oxygen species (ROS, e.g., superoxide and hydrogen peroxide), inducing the development of different mechanisms for iron homeostasis [19,49]. The regulation of iron uptake and storage is essential to maintain intracellular iron levels and fulfill metabolic needs across different environments. Iron is present in many proteins’ structures such as cytochromes, heme proteins, iron-sulfur (FeS) clusters, iron-molybdenum (FeMo) cofactors in nitrogenase, and nickel-iron (Ni-Fe) in hydrogenase [50,51].

##### Neodymium

Neodymium has no specific biological role, though in few available studies, Nd can assume different roles depending on its concentration and microbial species. In a recent study, it was shown to be used as a cofactor for biological methanol oxidation in the XoxF1-type MDH of the thermoacidophilic methanotroph *Methylacidimicrobium thermophilum* AP8, grown in a medium supplemented with neodymium as the sole lanthanide [46]. On the contrary, ammonia-oxidizing bacteria (AOB) were inhibited at a concentration above 20 ppm of Nd during ammonia wastewater treatment [52].

##### Tungsten

Tungsten is well known to induce the growth of methanogenic archaea, which produces methane using H_2_ and CO_2_ and is essential for many hyperthermophilic archaea growing at temperatures > 90° C [20]. A class of membrane-bound proteins involved in the tungsten import system named translocase has been reported. These translocases include ABC transporters, such as ModBC from *Archaeoglobus fulgidus* and ModABC, TupABC, and WtpABC transporter systems, which help microbial cells to uptake tungstate (WO_4_^2−^), which is subsequently converted into a tungstopterin cofactor [53,54]. Buessecker et al. provided an interesting study on the presence of tungsten membrane transport systems and pathways for anaerobic sugar oxidation mediated by tungsten-dependent enzymes by the anaerobic thermophilic archaea *Wolframiiraptor gerlachensis* [55]. Experimental results confirmed the dependence of *W. gerlachensis* growth on tungsten, particularly for carbohydrate metabolism involving tungsten-dependent ferredoxin oxidoreductases. Phylogenetic analyses of 78 *Wolframiiraptoraceae* genomes suggested the ancestral presence of tungsten-associated enzymes in this lineage [55]. These findings highlight the crucial role of tungsten in the metabolism, ecology, and evolution of this previously uncultivated archaeal lineage.

#### 2.1.5. Electrical Connectors

Wiring and micro-electrical parts contain copper, nickel, and precious metals like gold and silver used in the electrical connectors. The transition metal tantalum (Ta) is found in micro-capacitors for filtering and tuning frequency. Silicon metals are mainly used to manufacture processors due to its peculiar semiconductor nature along with other elements such as Ga, As, and Sb [48]. Electric wires could also contain tin and lead (Sn and Pb).

##### Silicon

The silicon (Si) element belongs to the carbon group. It is the second most abundant element in Earth’s crust due to the predominancy of silicate (SiO_4_)-based minerals. Silicon is an essential and beneficial element for very few microbes and organisms that implement biomineralization processes [19]. Some microorganisms are known to play a major role in the dissolution of minerals like silicates. In fact, the solubilization of insoluble silicon due to organic acid production by microbes is known to enhance silicate availability to plants [56]. In a case study on archaea, the hyperthermophile *Pyrococcus abyssi* was fossilized during and after exposure to a silica-saturated solution (about 500 ppm of SiO_2_) in a simulated hydrothermal environment [57], demonstrating its ability to bind silicon at the S-layer sites and integrate it in replacement of the cell wall. This phenomenon is also known as extracellular silicification, i.e., the process by which organisms incorporate on the cell surface soluble, monomeric silicic acid, Si(OH)_4_, in the form of polymerized insoluble silicon, SiO_2_. Moreover, some bacteria are also capable of uptaking silicic acid with a subsequent intracellular biosilicification [58].

##### Arsenic and Antimony

Both arsenic (As) and antimony (Sb) are useful for some selected microbes; they are commonly found as oxyanions and are subjected to biologically mediated redox reactions. Some microbes are able to oxidize or reduce arsenate for energy metabolism, playing a major role in the biogeochemical cycling of As [36]. Among archaea, *Sulfolobus acidocaldarius* was isolated in an acidic, sulfuric thermal spring in the Yellowstone National Park, being capable of oxidizing arsenite, As^4+^, to arsenate, As^5+^. Arsenite-oxidizing genes were also found in other archaea strains such as *Aeropyrum pernix* K1, *Pyrobaculum calidifontis* JCM 11548, and *Sulfolobus tokodaii* 7, and in the *Halorubrum* genus [59]. The oxidation of this metalloid can also support chemolithotrophic growth, with As^3+^ or Sb^3+^ serving as electron donors that provide reducing equivalents for the fixation of CO_2_ into organic matter, and as electron acceptors for anaerobic respiration [60]. The same happens for Sb with antimony-oxidizing bacteria that catalyze the oxidation of antimonite to the less-toxic antimonate. Interestingly, this process is considered as a potential efficient and environmentally friendly remediation technology for Sb pollution. With this mechanism, arsenic- and antimony-oxidizing bacteria can promote the release of As and Sb from ore deposits to the wider environment [60].

#### 2.1.6. Battery

Lithium-ion batteries (LIBs) are popular as main power supplies for portable devices, such as mobile phones and miniaturized electronics, because of the high energy density provided. LIBs consist of a cathode immersed in an electrolyte solution confined by a selective membrane and an anode of carbon material, commonly represented by graphite [61]. Common types of cathodes are lithium iron phosphate (LFP), lithium cobalt oxide (LCO), lithium manganese oxide (LMO), and lithium cobalt manganese/aluminium (NCM or NCA) [62]. In alkaline batteries (NCM), manganese (Mn) plays an important stabilizing role of the cathodic materials. Lithium batteries are constantly evolving along with new smartphone models, increasing the content of critical elements such as rare, precious, and toxic metals, with negative environmental implication [63].

##### Lithium

Li is one of the rarest among the light elements on Earth, its role in prokaryotes currently being under investigation [19]. A recent study highlighted the selective activation of gene expression induced by lithium riboswitches (preventing lithium toxicity particularly at high-pH culture conditions) [22]. For example, RNA elements that directly sense a physiological signal and, as a response, change their structure impact gene expression [64]. By recognizing metabolites, ions, or signaling molecules, riboswitches enable bacteria to adapt to changing environmental conditions and regulate essential biological processes, such as nutrient uptake, stress response, and virulence. These riboswitches play a crucial role in preventing lithium toxicity by controlling the expression of cation transporter genes, such as NhaA, which are involved in Na ion homeostasis and osmoregulation [65]. This research has implications for understanding bacterial response mechanisms against lithium toxicity and may have relevance in the context of increasing industrial applications of lithium in the biosphere. Cubillos et al., 2018, screened archaeal and bacterial communities present in the brines of Salar de Atacama [66]. In natural brines, the archaeal genera identified were *Halovenus*, *Natronomonas*, *Haloarcula*, and *Halobacterium*, while the most abundant bacterial families were *Rhodothermaceae* and *Staphylococcaceae*. Due to saline stress, hundreds of extremophile microorganisms developed special strategies to survive in lithium-rich brine. Moreover, lithium can compete with other cations such as Na^+^, K^+^, Mg^+2^, and Ca^+2^ in case of their shortage, due to its reduced ionic radius and high polarizing strength [67]. Jakobsson et al., 2017, provides insights into mechanisms similar to that of eukaryotic organisms for the uptake and utilization of lithium in bacterial systems [68]. The presence of lithium in living systems suggests that lithium had a significant impact on molecular and biological evolution. Understanding the interaction between microorganisms and lithium could support the development of biobased technologies addressing lithium recovery.

##### Cobalt

Cobalt is essential to the metabolism of many archaea and bacteria. In fact, Co is a key constituent of cobalamin, also known as vitamin B12. Vitamin B12 is an essential enzyme for all animals, but it can only be produced by bacteria and archaea. In ruminants, bacteria convert cobalt salts into cobalamin in rumen, whereas the other animals have to uptake cobalamin from diets. Recently, the presence of some metals, such as Co and Ni, have been reported to stimulate anaerobic digestion in methanogenic enriched cultures [69]. Cobalamin plays a crucial role in energy conservation in methanogenesis. The methyltransferase complex (MtrA-H) has in fact an important role as a methyl carrier from methyl-H_4_MPT to coenzyme M and it is proposed as a motor of the sodium ion pump [70,71].

##### Manganese

Mn is the twelfth most abundant element in Earth’s crust, and it is used by several living organisms, being essential for all photosynthetic bacteria and Eukarya. Conversely, several other bacteria have no documented need for Mn. In addition, we can find Mn as a cofactor in many metalloproteins, and, in some cases, it can be used as a substitute for iron in Fe-limited conditions [72]. Within the archaeal kingdom, members of the *Methanoperedenaceae* family were previously reported to conduct the iron/manganese-dependent anaerobic oxidation of methane (AOM), where Fe^3+^/Mn^4+^ constitute the terminal electron acceptors. Despite the potential for iron- and manganese-coupled AOM as a major methane sink in many Fe/Mn-rich sedimentary environments, the electron transport mechanisms that couple AOM with metal oxides are still under investigation.

**Table 1:** A summary of known metal–microorganism interactions in nature. Note that the role of metals is not necessarily associated with their extraction through bioleaching.

Element	Microorganisms	Element–Microorganism Interaction	References
*Bromine (Br)*	Methanogens	Inhibits methanogenesis by competitively binding key enzymes (e.g., methyl coenzyme M methyltransferase).	[25,26]
	*Asparagopsis* seaweed (source of bromoform)		
*Copper (Cu)*	Many bacteria and archaea	Essential cofactor in redox enzymes (e.g., ammonia monooxygenase). AOA require copper for ammonia oxidation.	[28,29,30,31]
	Ammonia-Oxidizing Archaea, AOA (e.g., *Nitrososphaera viennensis*)		
*Gallium (Ga)*	Limited microbial studies	Competes with iron in enzymes, disrupting iron-containing proteins (e.g., deoxyribonucleotide reductase).	[32,33,34]
*Germanium (Ge)*	Limited microbial studies	No established nutrient function.	[32]
*Nickel (Ni)*	Anaerobic bacteria and archaea	Essential in Ni enzymes (urease, Ni-Fe hydrogenase, methyl Co-M reductase). Key for methanogenesis (F_430_ cofactor).	[35,36]
	Methanogens (e.g., *Methanosarcina*, *Methanobrevibacter*)		
*Gold (Au)*	Thermophilic/hyperthermophilic bacteria and archaea (*Thermotoga maritima*, *Pyrobaculum islandicum*)	No known biological role; some microorganisms mediate Au solubilization/precipitation.	[37,38,39,40,41]
*Silver (Ag)*	Bacteria and archaea (general)	No known biological role: microorganisms can form Ag^+^ or Ag nanoparticles.	[37,38,40,41]
*Vanadium (V)*	*Methanosarcina mazei*, *Methanothermobacter thermautotrophicus*	Redox reaction V^5+^ → V^4+^ for detoxification or as an electron acceptor. Higher V reduction can inhibit methanogenesis.	[42]
*Niobium (Nb)*	No specific microorganisms cited	No known biological role in microorganisms.	[19]
*Tantalum (Ta)*	No specific microorganisms cited	No known biological role in microorganisms.	[19]
*Iron (Fe)*	Wide range of bacteria and archaea	Essential in heme proteins, Fe-S clusters, FeMo cofactors, and NiFe hydrogenases.	[19,49,50,51]
*Neodymium (Nd)*	Methylotrophs (*Methylacidimicrobium thermophilum* AP8)	Cofactor in XoxF-type methanol dehydrogenases (MDH). At high concentrations, Nd inhibits ammonia-oxidizing bacteria.	[46,52]
*Tungsten (W)*	Hyperthermophilic archaea (*Wolframiiraptor gerlachensis*)	Essential for tungsten-dependent oxidoreductases. Specialized ABC transporters (ModBC, TupABC, WtpABC) import tungstate.	[20,53,54,55]
*Silicon (Si)*	*Pyrococcus abyssi* (hyperthermophile)	Biomineralization (extracellular/intracellular silicification).	[19,56,57,58]
	Bacteria-solubilizing silicates		
*Arsenic (As)*	Arsenite-oxidizing archaea (*Sulfolobus acidocaldarius*, *Aeropyrum pernix* K1, *Halorubrum* sp.)	Redox reaction As^3+^ → As^5+^ for energy metabolism, aiding As detoxification.	[36,59,60]
*Antimony (Sb)*	Antimony-oxidizing bacteria (no species specified)	Oxidation of Sb^3+^ → Sb⁵^+^ for energy metabolism or detoxification.	[60]
*Manganese (Mn)*	Photosynthetic bacteria and eukaryotes *Methanoperedenaceae*	Essential cofactor in various metalloproteins. Anaerobic oxidation of methane (AOM) via Mn^4+^ reduction.	[72]
*Cobalt (Co)*	– Many bacteria and archaea– Rumen bacteria (convert Co salts to cobalamin)	Essential for cobalamin (vitamin B12) production; crucial in methanogenesis (methyltransferase Mtr complex). Presence of Co can stimulate anaerobic digestion in methanogenic cultures.	[69,70,71]
*Lithium (Li)*	– Halophiles (e.g., *Halovenus*, *Natronomonas*, *Haloarcula*, *Halobacterium*)	No confirmed essential role; Li riboswitches regulate cation transporter genes (e.g., *nhaA*).	[22,64,65,66,67,68]
	– Li riboswitches (bacteria)		

## 3. Microbial-Based Strategy for Critical Metal Recovery from EoL Mobile Phones

Extracting and recovering metals from e-wastes by exploiting microbial mechanisms can be seen as an analog of ore mining, in which microorganisms act as miners. Biohydrometallurgy is a biotechnological tool for mineral processing, which exploits the ability of microorganisms and their biogenic products, i.e., metabolites, to extract and concentrate metals from ores. Microbe–metal interactions occur through processes of metal immobilization and mobilization mechanisms. This process of the biological mobilization of metals is known as bioleaching [14,15,16,73].

**Figure 3:**
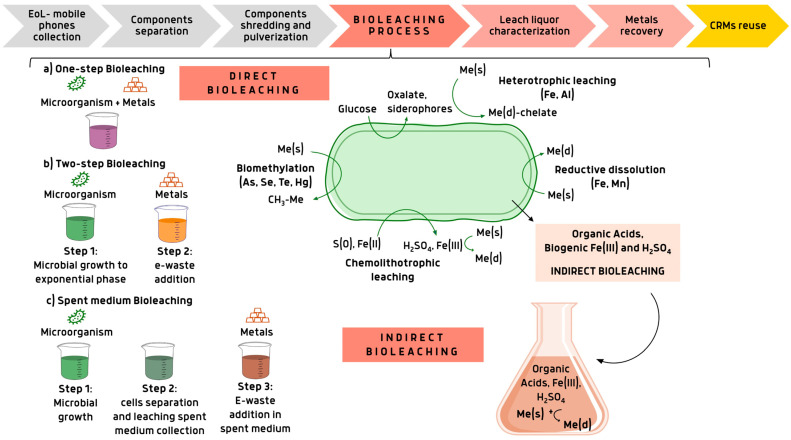
Flow diagram of EoL mobile phone recycling through bioleaching process and its different approaches. Abbreviations: Me (s): metal in solid state; Me (d): dissolved metal. Created via Canva.com (accessed on 23 September 2024).

Bioleaching relies on several biological mechanisms, which offer a unique approach to address the environmental challenges associated with traditional methods of metal extraction. Microbe–metal interactions occur through processes involving both metal immobilization and mobilization mechanisms. Microbes employ passive and active strategies to reduce metal toxicity, including metal influx, bioreduction, bioaccumulation-induced bioprecipitation (often involving polyphosphates), and biosorption. Additionally, microbes contribute to metal mobility through the production of organic acids during heterotrophic metabolism, reductive dissolution of oxides, and autotrophic oxidation of iron and sulfur in acidic environments [74]. Bioleaching processes are implemented through various mechanisms, broadly categorized as direct and indirect bioleaching. In direct bioleaching, microbial cells are placed in direct contact with e-waste powders. This contact can occur either at the beginning of the microbial growth process (one-step bioleaching) or after the microorganisms have reached the exponential growth phase (two-step bioleaching). The latter approach offers the advantage of allowing microorganisms to adapt and grow without being inhibited by the high concentrations of metals and toxic compounds present in the culture medium derived from e-waste powders. In both cases, cells facilitate metal interactions through the processes described earlier in this section, as illustrated in Figure 3. A third mechanism, known as indirect bioleaching, operates differently. In this process, microbial cells do not come into direct contact with e-waste. Instead, the interaction is mediated by microbial metabolites present in the spent microbial effluent, which is rich in organic acids and biogenic leaching agents such as Fe^3+^ or H₂SO₄. As shown in Figure 3, indirect bioleaching occurs in three steps: After microbial growth, the spent medium is collected by filtering out the microbial cells. The separated effluent is then used as leaching fluid to interact with e-waste [75].

**Figure 4:**
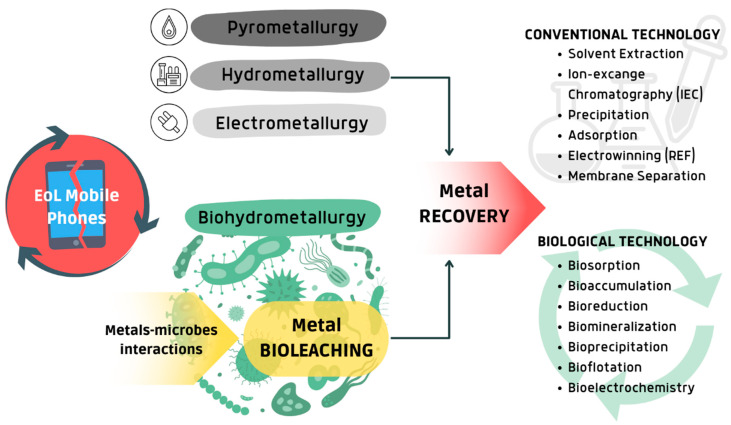
EoL mobile phone recycling in conventional versus biotechnological strategies. Created via Canva.com (accessed on 23 September 2024).

Compared to the conventional metallurgical techniques, bioleaching is considered effective in extracting metals when present in traces [76]. Bioleaching is often followed by the recovery of metals from the leachate solutions, an essential step in the overall process. Conventional metal recovery techniques include solvent extraction, ion exchange, precipitation, adsorption, and electrowinning [77,78]. These methods are generally well established and offer high recovery efficiency under controlled conditions. Despite their advantages, conventional techniques also have notable drawbacks. Solvent extraction and ion exchange often require large quantities of chemical reagents, raising concerns about environmental impact and operational costs [79]. Electrowinning is a clean, single-step process that does not generate secondary waste, making it environmentally advantageous compared to other metal recovery methods. However, it is energy-intensive, making scalability and process speed still challenging [80]. Precipitation and adsorption techniques, while relatively straightforward, sometimes lack selectivity, which can lead to the co-precipitation or adsorption of unwanted materials, thereby reducing purity and complicating downstream processes. Biological methods for metal recovery offer ecological alternatives to conventional techniques. Among them, biosorption, bioaccumulation, bioreduction, biomineralization, and bioprecipitation have already been widely discussed by the scientific community [15,76,81,82,83,84] (Figure 4).

Biosorption involves the passive binding of metal ions onto the surface of biological materials, such as algae, fungi, or bacteria, using cell wall components like polysaccharides and proteins. This method is cost-effective and operates under mild conditions, though some shortcomings can be the sensitivity to environmental factors, loss of efficiency due to the saturation of binding sites on the cell wall, and the need of metal desorption to regenerate microbial cells [85]. In contrast, bioaccumulation relies on the active uptake of metals by living organisms through metabolic processes, enabling high specificity but requiring nutrients to sustain the organisms and limiting its application in environments with high metal concentrations. Bioreduction employs microorganisms to enzymatically reduce metal ions to less toxic or insoluble forms. This approach is particularly effective for recovering metals, although it can be slow and sensitive to environmental conditions. Biomineralization and bioprecipitation involve the microbial-induced formation of stable mineral phases or metal precipitates, respectively, through processes like the production of hydrogen sulfide or phosphate. These methods are effective for stabilizing and recovering metals from mine waters, especially from dilute solutions, but often face challenges related to co-precipitation and scalability. Collectively, these biological techniques hold great promise for recovering valuable metals from electronic waste, mining leachates, and industrial wastewater while minimizing environmental impact [86].

However, selectivity remains a bottleneck for many methods, especially when dealing with complex metal mixtures. Thinking of smartphones as a whole mine compacted in a small space, the metals’ concentration in liquor remains a key parameter in building an efficient recovery strategy at the industrial scale.

### 3.1. Overview of Most Recent Bioleaching Studies Applied to Mobile Phones

**Figure 5:**
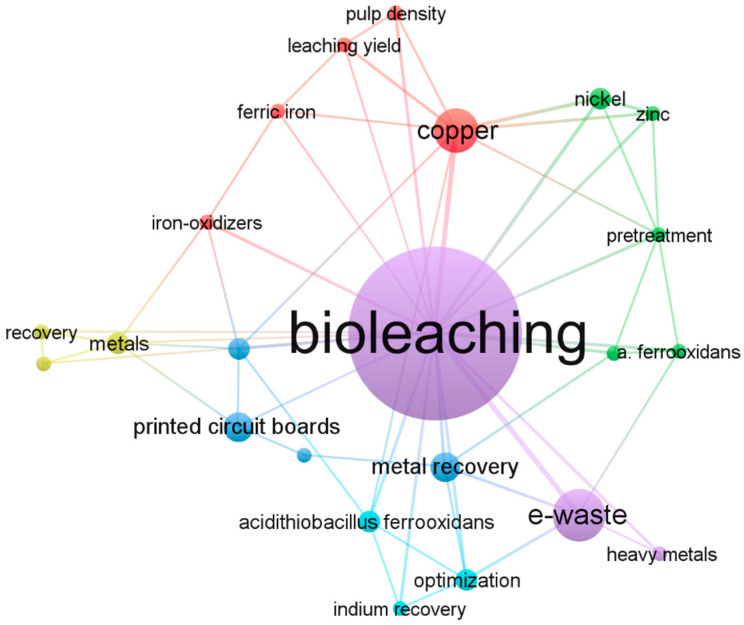
Network visualization map of most frequently used keywords along with clustering of keywords’ citation networks between 2018 and 2023 for mobile phone in bioleaching publications (minimum number of occurrences = 2).

To understand the weight of bioleaching in e-waste-related studies, a data set comprising works published between 2018 and 2023 was created (Table S1) using SciVal and Scopus platforms. The data set contained 261 papers, among which only 36 were related to mobile phone as a keyword. A graphical map of the authors’ keyword co-occurrence within these 36 studies is displayed using the non-commercial visualization of similarities viewer (VOSviewer, version 1.6.20; Figure 5). “Bioleaching” was found to be among the first 10 of the top 50 keywords for e-waste; electronic waste; and the Electronic Equipment Topic (Topic T.2195; https://www.scival.com/trends/keyphrases/wordcloud?uri=Topic/2195 accessed on 23 September 2024) and its relevance is growing over time, demonstrating the increasing interest in biohydrometallurgy as a sustainable alternative in modern techniques.

It is interesting to note that the word “copper” has almost the same occurrence as the word “e-waste”. Other frequently discussed metals within mobile phone-related publications include Fe, Ni, and precious ones, but almost no one considered the CRMs. Indeed, copper is the main element of PCBs, representing more than 60% of metals in these components and a remarkable fraction in overall mobile phone material. Another aspect to consider is the predominance of the “PCBs” word over other components in mobile phones, which is a trend already discussed by another recent study evidencing the main shortcomings in mobile phone studies [9]. As an example, among the 36 studies herein considered, only a few focused on bioleaching of screens [87,88,89]. Indeed, the word “indium recovery” appears in the network map (Figure 5). The keyword analysis also confirms that the acidophile bacteria *Acidothiobacillus ferroxindans* is the most popular species applied in bioleaching studies. The words “pulp density” and “leaching yield” occurred with similar frequency, consistently with the fact that, among key factors affecting the leaching efficiency, there is also the ratio between the e-waste and medium solution [90]. Moreover, by a search refining using “Consortium” as an author keyword, the exploitation of microbial consortia in the frame of bioleaching is discussed in 21 publications over the considered period (Table S1).

### 3.2. Metal Extraction Based on Microbial Consortia

Most bioleaching studies conducted so far have focused on pure cultures of *Acidothiobacillus ferroxindans* and *Acidithiobacillus thiooxidans*, Table 2 [88,91,92,93,94]. However, pure cultures can be outcompeted by mixed cultures depending on the consortia composition and metal content, Table 2 [95,96,97,98,99,100,101,102]. The advantage of using consortia and mixed cultures is due to broad metal tolerance held by different microorganisms of consortia, which can result in a higher metal resistance during leaching experiments [103]. In addition, the use of mixed cultures allows for a more robust and diverse microbial community, improving metal solubilization by leveraging synergistic effects of different microbial species. As demonstrated for some fungal cultures [104], non-conventional and economical growing medium, such as waste waters, can be also exploited to grow microbial consortia due to their high adaptability.

#### 3.2.1. Chemolithotrophic Acidophiles

Acidophilic bacteria such as *Acidithiobacillus ferrooxidans* and *Acidithiobacillus thiooxidans* are extensively employed in bioleaching due to their ability to thrive in acidic environments and their metabolic capacity to oxidize sulfur and iron compounds, which play a crucial role in dissolving metals.

Their primary roles include

- Oxidizing Fe^2+^ and reducing sulfur compounds to generate ferric iron (Fe^3+^) and sulfuric acid (H_2_SO_4_) [97], Table 2.
- Acidolysis and redoxolysis to dissolve base metals such as Cu, Zn, Al, and Ni.
- High pulp density tolerance (10–15% *w*/*v*) and less sensitivity to non-sterile conditions [95,101], Table 2.

Impressive copper recovery rates (98–99%) have been reported when acidophilic consortia are used for bioleaching of printed circuit boards (PCBs) from mobile phones [95,97,100,105].

Key representative species dominating the consortia often shift over time and include *Acidithiobacillus ferrooxidans* (Fe and S oxidizers) and *Leptospirillum ferriphilum* (Fe oxidizer) [101,106] followed by *Acidithiobacillus caldus* (S oxidizer), *Acidithiobacillus thioxidans* (S oxidizer) [107], *Sulphobacillus* sp. (Fe oxidizer), *Tissierella* sp. [95], and *Ferroplasma* sp. (archaeal species, Fe oxidizers) [107].

Despite the extractive efficiency, there are several constraints in using acidophile strains in bioleaching of mobile phones, and e-waste more in general:

- Some metals do not solubilize at low pH, at which they form insoluble salts [108].
- Acidophilic microorganisms require concentrated acid (H_2_SO_4_) and exterior S^0^ supplementation for their growth because they have optimal activity at a pH range of 2–3, but most e-wastes have an alkaline nature with theoretically zero sulfur content [109].
- Low-pH conditions risk corroding the process equipment and negatively impact the soil if the leachate is discarded in the environment.

#### 3.2.2. Heterotrophic Microorganisms

Heterotrophic microorganisms, including certain bacterial and fungal species, can facilitate bioleaching by producing metabolites such as organic acids and cyanogenic compounds, which help solubilize metals from mobile phone PCBs [97], Table 2. For instance, *Aspergillus niger* and *Penicillium simplicissimum* have been reported to achieve copper recovery rates of up to 65% [105]. In addition, using microbial consortia can enhance metal extraction, as demonstrated by the combination of *A. niger*, *Candida orthopsilosis*, and *Sphingomonas* sp., which yielded recovery rates of 54% for silver and 87% for gold [96,105]. Despite these promising results, the large-scale implementation of heterotrophic bioleaching is often hindered by the extensive accumulation of microbial biomass, which increases downstream processing demands and can elevate operational costs [87].

#### 3.2.3. Archaea (Thermoacidophiles and Methanogens)

Archaeal species can be extremely valuable due to their ability to thrive under harsh conditions (high temperature, extreme pH, high metal concentrations) [110,111]. Two main archaeal groups of interest include the following:

- Thermoacidophiles such as *Sulfolobus*, *Metallosphaera*, *Acidianus*, and *Sulfurisphaera* genera are chemolithotrophs that produce sulfuric acid and tolerate high metal concentrations [97,98,99,112].

Although not directly related to the case study of mobile phones, a number of *Sulfolobus* sp. including *Sulfolobus metallicus*, *S. acidocaldarius*, *S. solfataricus*, *S. brierly*, and *S. ambioalus* have been applied in several bioleaching studies [113,114]. *Sulfolobus metallicus* was adopted for bioleaching metals from a spent petroleum refinery catalyst, showing Ni and Al recovery ranging between 94 and 97%, and 54 and 59%, respectively [113]. Several extremophilic archaeon species can also remediate heavy metals such as As, Hg, and Cd as reported for the *Sulfolobus acidocaldarius* strain isolated from an acidic, sulfuric thermal spring in the Yellowstone National Park, which was found to be able to oxidize arsenite, As^4+^, to arsenate, As^5+^ [115], or as *Sulfolobus solfataricus* species capable of mercury, Hg^2+^, volatilization into Hg^0^ [116].

b. Methanogenic archaea: They produce methane via methanogenesis and can also recover critical platinum group metals (PGMs) via bioreduction and immobilization processes [117,118].

Other studies have instead employed single cultures of *Methanobacterium bryatii* BKYH, which is capable of chelating Cu^2+^ from Cu-rich mineral deposits [119], or *Methanothermobacter thermoautotrophicus* that could recover vanadium (V^4+^), chromium (Cr^3+^), and cobalt (Co^2+^) via bioreduction and immobilization processes [42,120,121].

While acidophiles dominate current applications, heterotrophic and archaeal species present promising alternatives that warrant further exploration. Overcoming limitations such as scalability, operational costs, and process integration is crucial for enhancing the feasibility of microbial methods in industrial e-waste recycling.

### 3.3. Biological Recovery of Metals from Leachate Solution

**Figure 6:**
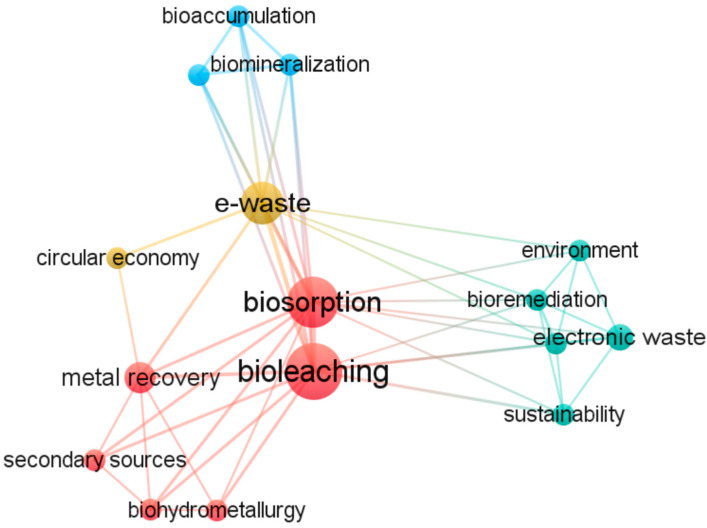
Network visualization map of most frequently used keywords in publications related to biosorption between 2018 and 2023 (minimum number of occurrences = 2).

Metal recovery within publications in e-waste; electronic waste; and the Electronic Equipment Topic for bioleaching is discussed in 109 publications. The word “metal recovery” comprises a wide range of meanings and does not refer only to the remediation or purification of metals from the leachate solution resulting from bioleaching but sometimes represents the bioleaching process itself. To seek relevant publications in the field of metal recovery, we restricted the search to the word “biosorption”, one of the main mechanisms in metal recovery after bioleaching; thus, only 17 studies were found (Table S2). Figure 6 shows the most used keywords within the publications involving biosorption studies, among which “bioaccumulation” and “biomineralization”, two further mechanisms of the recovery from leachate solutions, also occurred. Among other correlations, the word “bioremediation”, defined as a branch of biotechnology that exploits microorganisms to remove pollutants from contaminated environments, is closely related to “environment” and “electronic waste”, the extended definition of e-waste. “Sustainability” and “circular economy” are comprehensibly bound with the waste recovery issue, whose goal is to replace raw materials by using wastes as resources to fulfil a particular function [122].

#### Biosorption

Biosorption is a metabolism-independent mechanism that has been extensively researched for its effectiveness in removing heavy metals from industrial effluents [123]. In biosorption, microbial biomass binds metals to various cellular components such as cell walls, pigments, and extracellular polysaccharides (EPSs), whose production is triggered by the interactions between different microorganisms that enhance the overall biosorption capacity of the system [84,124,125]. Functional groups (e.g., OH, COOH, and NH_2_) on the microbial cells’ surface attach to metal ions dissolved in solutions. *Lactobacillus acidophilus* was utilized for biosorption in combination with ammonium thiosulphate as a chemical leaching agent to extract gold from waste PCBs, achieving an 85% recovery efficiency [126,127]. Fungi are also employed in biosorption: inactive biomass of *Aspergillus oryzae* (NCIM 1212) and baker’s yeast were successfully used for copper remediation from e-waste liquor, demonstrating a biosorption efficiency of 88.6% and 70.9%, respectively, under optimum reaction conditions [128]. Ambaye et al. reviewed the progress for REE recovery also using biosorption technology [129]. Researchers have investigated the recovery of lanthanum, neodymium, and cerium from e-waste using various biosorption methods. Lanthanum recovery has been performed by *Sargassum* biomass, *Pseudomonas* sp., and *Agrobacterium* sp. HN1. The biosorption of neodymium, mostly contained in permanent magnets, involved species such as *Monoraphidium* sp., baker’s yeast, *Penicillium* sp., *Saccharomyces cerevisiae*, *Kluyveromyces marxiamus*, *Candida colliculosa*, and *Debaryomyces hansenii*. Cerium biosorption was achieved using *Platanus orientalis* leaf powder and *Agrobacterium* sp. HN1. These methods have shown high efficiency and cost effectiveness in metal recovery. Factors influencing biosorption efficiency include the temperature, pH, agitation rate, contact period, and initial metal concentration. The compatibility of adsorbent technology and optimized process parameters is crucial for enhancing metal recovery from e-waste. Microbial consortia can exhibit synergistic effects, where different species complement each other’s abilities to enhance biosorption capacity and metal uptake rates. The diversity within a consortium can provide a wider range of functional groups and metabolic pathways, allowing for a more efficient and versatile metal removal process [130]. Although it is not part of the publication list selected for the present work, a relevant example in metal recovery is represented by microbial communities in bioelectrochemical systems applied for waste effluent treatment. Researchers have achieved the complete and selective reduction of chromium, copper, and cadmium thanks to the adaptation of bacterial communities at the cathodes, demonstrating the potential for microbial consortia to drive efficient metal remediation from wastewaters [131]. By harnessing the power of microbial communities, researchers have achieved significant improvements in recovery rates and selectivity of metals [132]. The use of microbial consortia in these processes shows potential for enhancing the efficiency and sustainability of metal recovery from waste materials.

## 4. Ecodesign as a Strategy for Enhancing the Biotechnological Recovery of Secondary Raw Materials from EoL Mobile Phones

**Figure 7:**
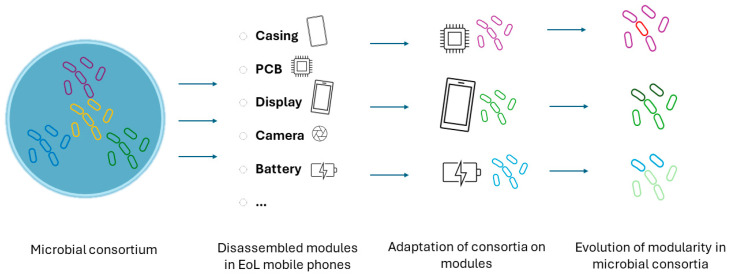
The interplay between modularity and specialization in microbial consortia adapting on individual components of EoL mobile phones during bioleaching.

Microbial consortia, whose benefits have been above described, can change and adapt to individual EoL mobile phone components, based on the different content in metals and metalloids. Modularity in electronic devices such as mobile phones means that individual components (e.g., battery, camera, usb ports, speaker units, etc.) can be individually dismantled and replaced. In the same way, modular structures are proper structures of biological systems (functional blocks) [133,134]. The interplay between specialization and modularity in biological systems plays a critical role in evolutionary adaptation and innovation by enabling the development of specialized functions, facilitating the evolution of new traits, and enhancing the microorganisms’ ability to thrive in diverse environments. Espinosa et al., 2010, highlighted how the modularity decreases the interference between different groups of genes, allowing for the maintenance of specialized functions and the evolution of new gene expression patterns specific to different body structures or environmental conditions [135]. In the frame of biotechnological recovery of secondary raw materials, a modular design could promote the selection of microbial communities, specifically targeting the components of EoL mobile phones (e.g., display, PCBs, speakers) during the bioleaching step (Figure 7). Ecodesign aims to integrate environmental aspects into the product development process, by balancing ecological and economic requirements throughout the entire product life cycle. In this regard, modular products may result in lowering environmental impact especially in the manufacturing phase [136]. The progressive research for the design for disassembly (DfD, i.e., the systematic assembly and disassembly of components) and modularity (i.e., the geometrical classification, which operates as a system arranged in a particular order) in electronics is intimately bound to biology [137]. Modular design relies on composing parts into devices, devices into circuits, and circuits into subsystems by “forgetting” the complexities internal to each device, circuit, or subsystem, once these are composed or inserted into a larger system [138].

### Europe Toward Ecodesign of Mobile Phones

Europe has prioritized resource efficiency, emission reductions, and strategic autonomy. A core focus is identifying alternative and circular sources of critical materials. New EU guidelines, directives, and official communications aim to support this transition by reshaping the manufacturing sector. The European Commission proposed the Ecodesign for Sustainable Products Regulation (ESPR) in March 2022 as part of the European Green Deal, to replenish the Ecodesign Directive 2009/125/EC by establishing essential requirements for sustainable product manufacturing (i.e., product upgradability, reparability, maintenance, and refurbishment). Currently, 29 product categories, including mobile phones, have been included within these ecodesign criteria. From 20 June 2025, mobile phones on the market should clearly present instructions on disassembly and repair, including responsibilities for producers to make critical spare parts available within 5–10 working days, and for 7 years after withdrawal from the market of the last unit of a product model [139]. These criteria are in harmony with the recent proposal for the directive “Right to Repair” as part of the 2020 Circular Economy Action Plan across the EU. The “Right to Repair” addresses the definition of common rules for product repair to make it easier and cost-effective for consumers, within and beyond the legal guarantee [140]. This combat planned obsolescence, extend product lifespans, and reduce electronic and other waste. Moreover, the new regulation on batteries set off, among others, the removability and replaceability as essential requirements of all batteries by 2027 [141]. The directive addresses the whole life cycle of batteries, including targets for the collection of waste batteries (63% by the end of 2027 and 73% by the end of 2030) and lithium recovery from them (50% by the end of 2027 and 80% by the end of 2031). Minimum levels of recycled content in batteries are also set at 16% for cobalt, 85% for lead, 6% for lithium, and 6% for nickel. The EU identifies biotechnology and life sciences as key to tackling environmental, social, and economic challenges. Biotechnological solutions can reduce reliance on fossil-based and critical raw material streams, aligning with broader goals of resource independence and environmental sustainability (https://ec.europa.eu/commission/presscorner/detail/en/ip_24_1570, accessed on 23 September 2024). Consistently with these proceedings by the European Commission, the co-funded EU Sustronics project is attempting to revolutionize the European electronics industry by focusing on green technologies. The project aims to introduce sustainable manufacturing concepts using biogenic materials to enhance the self-sufficiency of raw materials’ supply chain. Concerning biobased materials, the PRiNGLE project (grant agreement No. 101046719) addresses the design of a novel class of protein materials evolved by marine cable bacteria [142], which own high conductive features that allow their integration in electronic devices [143].

## 5. Conclusions

The present review highlights the complexity of mobile phones in terms of structure and material composition, along with the potential interactions that specific microorganisms can establish with these materials. Many metals essential in mobile phone manufacturing play specific biological roles in microorganisms, opening the door to sustainable recovery strategies for critical and strategic raw materials through biotechnological methods. Bioleaching and biorecovery represent the key biological mechanisms that enable metal mobilization and immobilization, offering a viable alternative to traditional energy-intensive recycling methods. Although 261 bioleaching-related studies between 2018 and 2023 have been identified, among these, only 36 are dedicated to mobile phones. Moreover, research should expand beyond PCBs to address underexplored components such as vibration units, cameras, and casings, which also contain critical raw materials. Complexity, which is proper to electronic devices, can be faced by the synergy of microorganisms, which own the intrinsic ability to thrive in diverse environments by developing specific functions for specific layers. Investigating the synergistic effects of microbial consortia in targeting multi-metal layers within complex components will further enhance bioleaching efficiencies. Future research should focus on optimizing microbial consortia capable of selectively targeting specific components of mobile phones, such as screens, batteries, or PCBs. Strategies to minimize the generation of sludge and other toxic byproducts during bioleaching processes must be developed. Moreover, enhancing the understanding of biosorption, bioaccumulation, and bioprecipitation as secondary recovery mechanisms will effectively close the material loop. In this regard, we also highlight the importance of aligning bioleaching advancements with the principles of the circular economy. By targeting critical metals, these methods can reduce reliance on virgin raw materials and promote sustainable practices within the electronics industry. Furthermore, the implementation of EU directives on ecodesign and recycling, such as the Critical Raw Materials Act and Ecodesign regulation, represents a unique opportunity for industry stakeholders to lead in sustainability efforts. While bioleaching technologies are still emerging, their potential for scaling up industrial applications is immense. Through collaboration between academia, industry, and policymakers, these biotechnological solutions can contribute significantly to sustainable resource recovery, environmental protection, and the establishment of a robust circular economy framework.

## Figures and Tables

**Table 2:** Summary of bioleaching studies on EoL mobile phones and/or e-waste-containing mobile phone components, using pure culture or mixed culture or microbial consortia.

Bioleaching Condition	Culture Type	Metal Leaching (% or mg)	Reference
Optimal conditions (ferrous sulfate: 13.0 g/L; touch screen content: 3.0 g/L; elemental sulfur: 5.6 g/L; and initial pH of 1.1)	Adapted *Acidithiobacillus ferrooxidans* pure culture	100% In (200 mg/L indium); 5% Sr (3000 mg/L)	[88]
Indirect two-step bioleaching process: Step 1—Fe^2+^ bio-oxidation to Fe^3+^ by *Acidithiobacillus ferrooxidans* (48 h); Step 2—Fe^3+^ used for Cu solubilization; 7.5 g/L PCB, 48 h total,	*Acidithiobacillus ferrooxidans* pure culture	Cu: 95–100%	[91]
15 g/L pulp density, 10% (*w*/*w*) inoculum size, 30 °C, 130 rpm, mixed e-waste	Adapted *Acidithiobacillus* *ferrooxidans* pure culture	Cu: 100%, Fe: 100%, Ni: 54%	[92]
0.5 mm particle size e-waste, variable pulp densities (0.5%, 1%, 1.5%, 2%)	*Acidithiobacillus ferrooxidans* and *Acidithiobacillus thiooxidans* pure culture	Cu: 79% (*A. ferrooxidans*, 1% pulp density), Ni: 80%, Al: 70% (*A. ferrooxidans*, 0.5% pulp density), Co: 61.7%, Zn: 60.9%, Pb: 49.8% (*A. thiooxidans*, 0.5% pulp density), Au: 55% (*A. ferrooxidans*, 1% pulp density), Au: 67% (*A. thiooxidans*, 1% pulp density)	[93]
9K medium, 35 days for In, 14–21 days for Sn	Mixed adapted bacteria (*Acidothiobacillus ferrooxidans* and *Acidothiobacillus thiooxidans*)	In: 55.6%; Sn: 90.2%	[94]
batch bioleaching at varying pulp densities of 7%, 10%, and 15% (*w*/*v*). Cu content in the feed material was 26.3% (*w*/*w*)	Mixed microbial consortia of iron- and sulfur-oxidizing microorganisms	Cu: 98–99%	[95]
pH 4.5 (initial), decreased to 2.8 (in consortium), 35 days, glucose as carbon source, no agitation	(a) *Aspergillus niger* MXPE6 pure culture, (b) Fungal Consortium	(a) Au 17%, (b) Au 56%	[96]
Multi-metal extraction from PCBs and tantalum capacitor scrap	(a) Mixed consortium of acidophiles and heterotrophic fungal strains (b) Pure *A. niger* filtrate containing sulfuric, citric, and oxalic acids	(a) PCB samples: Ni and Cu (99% and 96%, respectively); Fe, Zn, Al, and Mn (89, 77, 70, and 43%, respectively). Tantalum capacitor samples: 92% Cu, 88% Ni, 78% Fe, 77% Al, 70% Zn, and 57% Mn. (b) PCB samples: Cu, Fe, Al, Mn, Ni, Pb, and Zn at an efficiency of 52, 29, 75, 5, 61, 21, and 35%. Tantalum capacitor samples: 61, 25, 69, 23, 68, 15, and 45% from tantalum capacitor samples, respectively.	[97]
pH of 10.0, pulp density of 5 g/L, and leaching time of 34 h	Mixed culture of cyanide-producing strains of *Pseudomonas putida* and *Bacillus megaterium*	Au: 83.59%	[98]
pH 1.8; initial ferrous concentration of 9 g L^−1^; 4–7 days; pulp densities of 10, 50, and 100 g L^−1^; acidophilic iron oxidizers	Iron-oxidizing microbial consortium	Cu: 275 mg (10 g L^−1^), 1350 mg (50 g L^−1^), 2640 mg (100 g L^−1^); Zn: 5 mg (10 g L^−1^), 18 mg (50 g L^−1^), 25 mg (100 g L^−1^); Ni: 11 mg (10 g L^−1^), 53 mg (50 g L^−1^), 100 mg (100 g L^−1^)	[99]
9 g/L Fe^2+^, 10% pulp density, initial pH 1.8, 10% (*v*/*v*) initial inoculum, ORP > 750 mV (shake flask) and >650 mV (bench-scale bioreactor), 8 days	Mixed meso-acidophilic bacteria	Cu: 98.1%, Al: 55.9%, Ni: 79.5%, Zn: 66.9% (shake flask); Cu: 97.3%, Al: 55.8%, Ni: 79.3%, Zn: 66.8% (bench-scale bioreactor)	[100]
Indirect bioleaching with ferric iron lixiviant at constant pH (2)	*Leptospirillum*-dominated consortium	Cu at 96.86% from NaOH pretreated unpulverized PCBs; Zn at 90.69% from NaOH pretreated pulverized PCBs; Ni at 93.65% with untreated unpulverized PCBs	[101]
pH: acidic and alkaline, 10 g/L e-waste loading, indigenous acidophilic heterotrophic bacterial consortium sourced from iron ore soils	Indigenous acidophilic heterotrophic bacterial consortium	Cu: 4%, Cr: ≤0.002%, Overall: 4.7%	[102]

## Data Availability

All the data generated within the present study are available in the Supplementary Materials.

## Supplementary Materials

The following supporting information can be downloaded at: https://www.mdpi.com/article/10.3390/bioengineering12020101/s1. Table S1: Publications in E-Waste; Electronic Waste; Electronic Equipment for Bioleaching; Table S2: Publications for Biosorption within Publications in E-Waste; Electronic Waste; Electronic Equipment for Bioleaching.
